# Postpartum Depression and Its Biological Biomarkers

**DOI:** 10.7759/cureus.31124

**Published:** 2022-11-05

**Authors:** Arya Rathi, Shrutika Khapre, Jay Chavada, Saloni Gupta, Tanvi Singla

**Affiliations:** 1 Obstetrics and Gynecology, Jawaharlal Nehru Medical College, Datta Meghe Institute of Medical Sciences, Wardha, IND

**Keywords:** postpartum women, postpartum care, women's mental health, mother and child health, postnatal depression, postpartum depression, psychotherapy, biomarkers, hpa axis, depression

## Abstract

A woman's life is significantly impacted by both pregnancy and childbirth. A woman's tasks and obligations undergo abrupt and significant adjustments as a result of having a child. As a result, the postpartum period is when a new mother is most likely to develop postpartum depression. It frequently has serious detrimental effects on the infant. Similar signs and risk factors can also be seen in non-postpartum depression. The main difference is that postpartum-specific factors, including biological and psychosocial ones, are what lead to postpartum depression. Among biological processes, inflammatory processes and hypothalamic-pituitary-adrenal dysfunction are the best indicators of postpartum depression risk. Many biomarkers have also been discovered using the cutting-edge multi-omics approach. Psychotherapy and antidepressants are frequently used to treat postpartum depression, although there has been much worry about the drugs' potential negative effects, such as decreased appetite, dizziness, headaches, and drowsiness. To prevent the negative effects of postpartum depression on both mother and child, it is crucial to correctly identify and treat it during the postnatal period as soon as feasible.

## Introduction and background

Most moms experience excitement and happiness with the birth of their child, but postpartum depression (PPD) affects many new mothers [1]. According to estimates, postpartum depression may affect 15% of women during the first year after giving birth [2]. PPD is an incapacitating disorder, much like serious depression. Suicide related to PPD is the second-leading cause of mortality for postpartum women [3]. Postpartum depressive symptoms (PDS) can arise up to a year after childbirth and can peak as early as four to six weeks after delivery. They typically go away on their own between two and six months after giving birth, although they might remain longer [4]. It can be perplexing and upsetting to experience the tension between the happy emotions that new moms frequently believe they should feel and the depressive mood and worry that many of them experience. Women could believe that these symptoms will just go away by themselves and won't require any treatment, as is generally the case for postpartum blues, a milder type of mood disorder that happens within the first ten days of giving birth [5]. Many manifestations, including melancholy, nausea, anxiety, irritability, feelings of loneliness, and abnormalities in sleep, can be present with PPD [6,7]. Another common sign of PPD that can harm family relationships is having thoughts of harming oneself or the baby [8-10]. A woman goes through physiological changes during pregnancy, labor, and lactation. Sadly, a woman's vulnerability to mental illness is greatest during the perinatal period. PPD is under-recognized and under-treated. The obstetrician and pediatrician can serve important roles in screening for and treating PPD. Postpartum depression interferes with a person's psychological and mental health; an incomplete understanding of its etiology constitutes a barrier to early identification and treatment [11]. The objective of this article is to understand the etiology of postpartum depression and its identification using different biomarkers to identify it as early as possible and treat it.

## Review

The diagnosis of PPD

The diagnosis of PPD can be done in several ways. The Diagnostic and Statistical Manual of Mental Disorders, Fifth Edition, should be used to guide any interview design [12]. Additionally, self-report tools like questionnaires have been extensively employed in clinical evaluation. Any interview design must be based on the Diagnostic and Statistical Manual of Mental Disorders, Fifth Edition [13]. However, using only such arbitrary scales could result in subjective bias. The most effective approach in clinical practice is to combine these subjective evaluations with the application of some objective markers to make a better diagnosis [14]. As a result, the profile of the endocrine system is taken into account, together with clinic interviews and self-rating scales, in addition to hormones of the hypothalamic-pituitary-adrenal (HPA) axis [15,16]. The HPA axis is frequently dysregulated in people with severe depression and PPD [17,18]. Because the psychological changes moms go through could be a particular system for controlling stress and parturition, three key HPA axis hormones, corticotrophin-releasing hormone (CRH), adrenocorticotropic hormone (ACTH), and cortisol, have been well studied in prenatal and postnatal depression [19-21]. The concentration of a hormone is a reliable predictor of a patient's neuroendocrine health, which is just one advantage of using hormones as biomarkers. The sampling method can easily be standardized and integrated into nurses' and technicians' routine duties. Furthermore, numerous biomarkers can be investigated simultaneously in a single sample. Thirdly, there are several potential applications for prenatal blood stress hormone screening, including monitoring the control and metabolism of stress during pregnancy, screening for depression or anxiety during pregnancy, and anticipating preterm labor and the commencement of delivery. The participant's socioeconomic status and demographics, which are essential for predicting the development of PPD, are not revealed by biomarker assessment alone, though. As a result, combining a hormone study with a conventional behavioral assessment is preferred. [22,23]. Certain scales used for depression are the postpartum depression screening scale, the two-item patient health questionnaire (PHQ-2), the nine-item patient health questionnaire (PHQ-9), and the Hamilton Rating Scale for Depression [24,25]. 

Various Models Of Postpartum Depression

**Figure 1:**
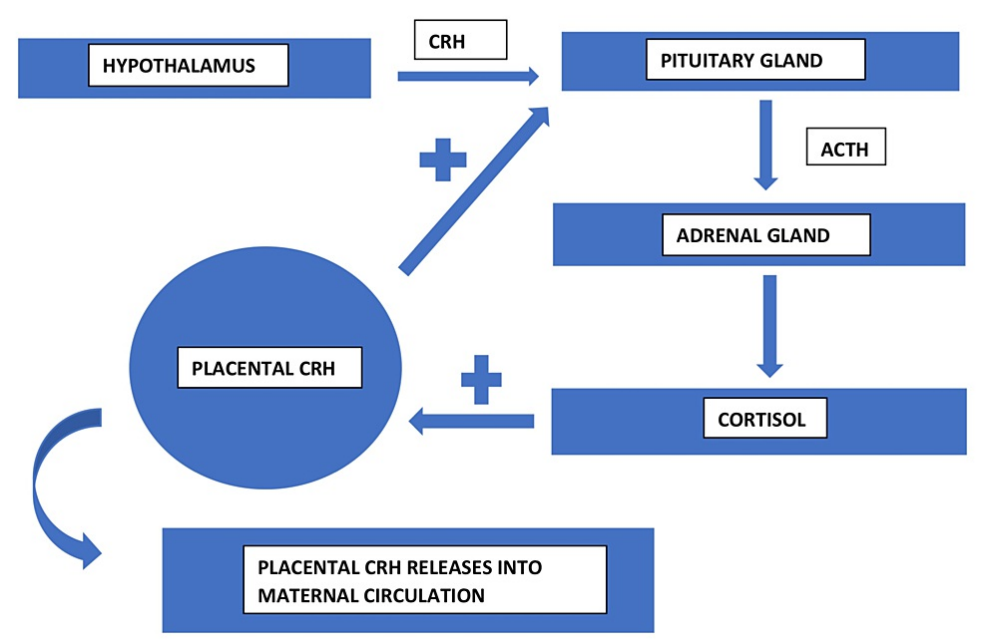
HPA axis in pregnancy CRH = corticotropin-releasing hormone ACTH = Adrenocorticotropic hormone

HPA-Axis: The HPA axis contains numerous hormones that control the release of glucocorticoids, primarily cortisol, in people. Although the HPA axis is often associated with the production of cortisol in response to stress, cortisol also regulates essential basic homeostatic functions in addition to responding to immediate stressors (metabolism and immune system regulation [26]. Pregnancy, childbirth, and lactation all significantly modify how the HPA axis and circulating cortisol operate. Placental CRH (pCRH), to some extent, is in charge of important pregnancy phases like nursing, pregnancy, and birthing, which result in high cortisol levels and numerous essential basic and unique functional overlaps between the HPA hormones (Figure 1) [27]. Although there is a lot of individual variation, the last few weeks of pregnancy are when cortisol levels rise the steepest, reaching levels that are three times higher than those of non-pregnant women [28].

The greater basal cortisol concentrations in human pregnancy are primarily due to the placenta rather than the HPA axis. Throughout pregnancy, the placenta gradually assumes the role of the endocrine gland. Besides estrogen and progesterone, the placenta also produces pCRH, which is similar to hypothalamic CRH in bioactivity and structure [29]. The increase in circulating cortisol levels is caused by pCRH. The human placenta is stimulated by pCRH and the ensuing rise in cortisol levels to generate pCRH. The subsequent stimulation of cortisol production by pCRH creates a positive feedback loop [30,31]. The cortisol level in women cannot be very high, even though levels of cortisol during pregnancy often exceed those seen in healthy, non-pregnant people. When the fetus is exposed to ideal and adaptable circulating cortisol concentrations, it is simpler for it to grow and develop, which also aids in maintaining pregnancy. Additionally, the HPA axis' responsiveness to stress is suppressed at the time of pregnancy and in the postpartum period. This diminished HPA axis response works to protect the mother and newborn [32]. After a vaginal delivery as opposed to a C-section, there is a noticeable rise in cortisol levels during parturition. An increase in the level of cortisol may be a reaction to how difficult the labor was, and it is probably necessary to get the baby ready for the outside world. The source of pCRH is thereafter eliminated with the delivery of the placenta. Within 15 hours of delivery, plasma CRH concentrations revert to normal levels from before conception. In the days and weeks following delivery, cortisol also significantly declines, which helps to significantly change how the HPA axis is regulated [33]. Women with significant depression had reduced cortisol stress responses. The dysregulation of the HPA axis is frequently linked to mental disease in both males and females [34,35]. According to Kammerer et al., although depression after childbirth may be defined by low cortisol levels, depression during pregnancy may be characterized by high cortisol levels [36]. They also proposed that depression would be more melancholy during pregnancy and more atypical throughout the postpartum period. According to O'Connor et al., major depressive disorder during pregnancy is associated with a lower level of cortisol at awakening compared to control groups but significantly higher cortisol levels overall [37]. Significant mood symptoms in pregnancy have been linked to changed diurnal cortisol levels, which have been proven to be reliable biomarkers of anxiety and depression. According to Glynn et al., PPD may be predicted by prenatal HPA axis dysregulation, with higher or accelerated pCRH trajectories throughout gestation being linked to an enhanced postpartum decline in cortisol [38,39].

Reproductive Hormones

The timing of pregnancy, labor, and delivery is significantly influenced by reproductive hormones.

Progesterone

Progesterone level and PPD level appear to have a strong connection, according to studies. It was discovered that the levels of allopregnanolone (ALLO) gradually rose during gestation before sharply declining after delivery. ALLO is a progesterone metabolite that is a neuroactive steroid that can be detected in the peripheral circulation. Because of this, its levels fluctuate in proportion to progesterone levels both throughout pregnancy and after delivery. Additionally, a link between ALLO and PPD was discovered, indicating that hormonal modulation is crucial for the development of PPD [40-42]. Bloch et al. found that PPD patients are more vulnerable to the mood-stabilizing effects of gonadal steroids than healthy controls. Low ALLO levels during pregnancy accurately predict PPD rather than progesterone withdrawal after delivery [43,44].

Oxytocin

The oxytocin signaling network has attracted a lot of attention because it can be crucial for interactions and bonding between mothers and their infants. Lower oxytocin levels are linked to an increased risk of PPD development in both the gestational and postpartum periods [45,46]. According to Jobst et al., from week 35 of pregnancy to six months after giving birth, plasma oxytocin levels in all women significantly increased. However, levels declined in women with PPD from the 38th week of pregnancy to two days after birth, whereas they constantly rose in the healthy control group. This suggests that the time evolution pattern of oxytocin can predict PPD in the immediate postpartum period (within two weeks) [47].

Thyroid Hormone

In certain instances, thyroid autoimmunity-related thyroid dysfunction is linked to the physiological changes that occur after birth, serving as a predictor for PPD. There is evidence that thyroid hormone (TH), which has been associated with severe neurological impairments, may enhance the risk of PPD due to its aberrant expression in the early postnatal period [48-50]. According to Kuijpens et al., the presence of thyroperoxidase antibody (TPOAb) at the time of pregnancy is linked to the development of depression in the postpartum period; as a result, it can be used as a marker for postpartum depression. Six months after giving birth, elevated thyroid stimulating hormone (TSH) has been suggested to be a predictor of PPD [51,52]. According to Li et al., estrogen, progesterone, and TSH levels were lower in PPD patients, whereas triiodothyronine, thyroxine, free triiodothyronine, and free thyroxine levels were greater. Thyroid hormones play a role in the genesis of PPD, but it has also been suggested that they may work best when combined with other factors like estrogen levels or a history of trauma [53,54]. Studies linking PPD and thyroid function suggest [55,56] that thyroperoxidase antibodies (TPOAb) may be a potential target in the search for a biomarker to predict the development of emotional disorders, including PPD.

Inflammatory Markers

A growing amount of literature indicates that the pathophysiology of depression is significantly influenced by inflammatory responses [57]. Proinflammatory cytokine levels in puerperal women are shown to be much higher in the final trimester of pregnancy, and they are also likely to experience depression. Stress can be reduced and the inflammatory response can be controlled when breastfeeding. In general, a pro-inflammatory state throughout late pregnancy and the early postpartum period is crucial to the emergence of PPD [58]. The most often examined cytokine, interleukin-6 (IL-6), has repeatedly been found to be increased in depressive disorders. According to Corwin et al., PPD patients had higher levels of interleukin-1 (IL-1) on days 14 and 28 postpartum compared to euthymic women, indicating a link between PPD symptoms and elevated levels of IL-1 during the first month after delivery [59]. Liu et al. observed that PPD development during the first six months after delivery was linked to higher serum IL-6 levels [60]. TNF ligand superfamily member (TRANCE), hepatocyte growth factor (HGF), IL-18, fibroblast growth factor 23 (FGF 23), and C-X-C motif chemokine 1 (CXCL1) were all found to be significantly higher in PPD-affected women [61].

Biochemical Markers

Identifying biochemical and dietary indicators for PPD diagnosis has gained more attention recently. In PPD patients, on the third postpartum day, According to Wójcik et al., a decline in serum zinc level and the severity of depression symptoms are related [62]. According to Roomruangwong et al., lower blood zinc levels and higher CRP levels were found to be significantly predictive of prenatal depression and physio-somatic symptoms, which together were found to be substantially predictive of postnatal depressive symptoms [63]. According to Christesen et al.'s study on the effects of vitamin D on pregnancy, low levels of vitamin D during pregnancy may contribute to PPD and preeclampsia in many studies [64]. Brandenbarg et al. found a correlation between lower early-pregnancy vitamin D levels and increased depressive symptoms during pregnancy [65]. Gur et al. found a link between lower maternal 25(OH)D3 levels and higher PPD levels over time, indicating that these levels may play a role in the development of PPD [66]. PPD symptoms are more likely to develop when vitamin D levels are low during pregnancy [67]. Women with PPD were significantly more likely to have lower levels of vitamin D during the prenatal period than women without, according to an exploratory study of antenatal vitamin D levels [68].

## Conclusions

Both the mother, who has maternal postnatal depression, and her infant up to the age of three, suffer detrimental effects as a result of this disease. Due to its high incidence and the pain it causes, PPD requires urgent early detection and treatment. The endeavor to diagnose PPD using predictors from both psychological and biological factors has increased. However, biochemicals may also serve as the appropriate signs or indications that can be employed to detect and anticipate PPD. Preliminary investigations have identified several hormones, neuro-steroids, and biochemicals as promising biomarkers for predicting PPD; however, more research and substantiation are required before they can be used clinically. The development of children appears to be more at risk from chronic maternal depression than from less severe depression. As a result, maternal PPD has a variety of detrimental direct and indirect impacts on a child's development, including poorer quality of the home environment and diminished maternal sensitivity and caregiving. To prevent negative effects, it seems crucial to identify and treat depression in the postnatal period as soon as feasible. The ultimate objective of researching numerous PPD predictors and biomarkers is the early detection and correction or avoidance of such disorders.

## References

[1] Gavin NI, Gaynes BN, Lohr KN, Meltzer-Brody S, Gartlehner G, Swinson T (2005). Perinatal depression: a systematic review of prevalence and incidence. Obstet Gynecol.

[2] Yim IS, Dunkel Schetter C (2019). Biopsychosocial predictors of perinatal depressive symptoms: moving toward an integrative approach. Biol Psychol.

[3] Serati M, Carnevali G: Perinatal Depression. In: Altamura AC, Brambilla P, eds eds (2019). Perinatal depression. Clinical Cases in Psychiatry: Integrating Translational Neuroscience Approaches.

[4] Lee DT, Chung TK (2007). Postnatal depression: an update. Best Pract Res Clin Obstet Gynaecol.

[5] Grigoriadis S, Romans S (2006). Postpartum psychiatric disorders: what do we know and where do we go?. Curr Psychiatry Rev.

[6] Wang Z, Liu J, Shuai H (2021). Correction: mapping global prevalence of depression among postpartum women. Transl Psychiatry.

[7] Robertson E, Grace S, Wallington T, Stewart DE (2004). Antenatal risk factors for postpartum depression: a synthesis of recent literature. Gen Hosp Psychiatry.

[8] Howard LM, Flach C, Mehay A, Sharp D, Tylee A (2011). The prevalence of suicidal ideation identified by the Edinburgh Postnatal Depression Scale in postpartum women in primary care: findings from the RESPOND trial. BMC Pregnancy Childbirth.

[9] Orsolini L, Valchera A, Vecchiotti R (2016). Suicide during perinatal period: epidemiology, risk factors, and clinical correlates. Front Psychiatry.

[10] Wisner KL, Sit DK, McShea MC (2013). Onset timing, thoughts of self-harm, and diagnoses in postpartum women with screen-positive depression findings. JAMA Psychiatry.

[11] Pearlstein T, Howard M, Salisbury A, Zlotnick C (2009). Postpartum depression. Am J Obstet Gynecol.

[12] Austin MP (2010). Classification of mental health disorders in the perinatal period: future directions for DSM-V and ICD-11. Arch Womens Ment Health.

[13] Eberhard-Gran M, Eskild A, Tambs K, Opjordsmoen S, Samuelsen SO (2001). Review of validation studies of the Edinburgh Postnatal Depression Scale. Acta Psychiatr Scand.

[14] Asakawa T, Fang H, Sugiyama K (2016). Human behavioral assessments in current research of Parkinson's disease. Neurosci Biobehav Rev.

[15] Gordon JL, Girdler SS, Meltzer-Brody SE (2015). Ovarian hormone fluctuation, neurosteroids, and HPA axis dysregulation in perimenopausal depression: a novel heuristic model. Am J Psychiatry.

[16] Garcia-Leal C, De Rezende MG, Corsi-Zuelli FM, De Castro M, Del-Ben CM (2017). The functioning of the hypothalamic-pituitary-adrenal (HPA) axis in postpartum depressive states: a systematic review. Expert Rev Endocrinol Metab.

[17] Lloyd RB, Nemeroff CB (2011). The role of corticotropin-releasing hormone in the pathophysiology of depression: therapeutic implications. Curr Top Med Chem.

[18] Rothe N, Steffen J, Penz M, Kirschbaum C, Walther A (2020). Examination of peripheral basal and reactive cortisol levels in major depressive disorder and the burnout syndrome: a systematic review. Neurosci Biobehav Rev.

[19] Vitoratos N, Papatheodorou DC, Kalantaridou SN, Mastorakos G (2006). "Reproductive" corticotropin-releasing hormone. Ann N Y Acad Sci.

[20] Payne JL, Maguire J (2019). Pathophysiological mechanisms implicated in postpartum depression. Front Neuroendocrinol.

[21] Valsamakis G, Chrousos G, Mastorakos G (2019). Stress, female reproduction and pregnancy. Psychoneuroendocrinology.

[22] Ahn S, Corwin EJ (2015). The association between breastfeeding, the stress response, inflammation, and postpartum depression during the postpartum period: prospective cohort study. Int J Nurs Stud.

[23] O'Keane V, Lightman S, Patrick K (2011). Changes in the maternal hypothalamic-pituitary-adrenal axis during the early puerperium may be related to the postpartum 'blues'. J Neuroendocrinol.

[24] Stickel S, Eickhoff SB, Habel U, Stickeler E, Goecke TW, Lang J, Chechko N (2021). Endocrine stress response in pregnancy and 12 weeks postpartum - Exploring risk factors for postpartum depression. Psychoneuroendocrinology.

[25] Zhao Y, Kane I, Wang J, Shen B, Luo J, Shi S (2015). Combined use of the postpartum depression screening scale (PDSS) and Edinburgh postnatal depression scale (EPDS) to identify antenatal depression among Chinese pregnant women with obstetric complications. Psychiatry Res.

[26] Joseph DN, Whirledge S (2017). Stress and the HPA axis: balancing homeostasis and fertility. Int J Mol Sci.

[27] Chai Y, Li Q, Wang Y, Tao E, Asakawa T (2022). The value of HPA axis hormones as biomarkers for screening and early diagnosis of postpartum depression: updated information about methodology. Front Endocrinol (Lausanne).

[28] Soma-Pillay P, Nelson-Piercy C, Tolppanen H, Mebazaa A (2016). Physiological changes in pregnancy. Cardiovasc J Afr.

[29] Lindsay JR, Nieman LK (2005). The hypothalamic-pituitary-adrenal axis in pregnancy: challenges in disease detection and treatment. Endocr Rev.

[30] King BR, Nicholson RC, Smith R (2001). Placental corticotrophin-releasing hormone, local effects and fetomaternal endocrinology. Stress.

[31] Robinson BG, Emanuel RL, Frim DM, Majzoub JA (1988). Glucocorticoid stimulates expression of corticotropin-releasing hormone gene in human placenta. Proc Natl Acad Sci U S A.

[32] Jones SA, Brooks AN, Challis JR (1989). Steroids modulate corticotropin-releasing hormone production in human fetal membranes and placenta. J Clin Endocrinol Metab.

[33] Brunton PJ, Russell JA, Douglas AJ (2008). Adaptive responses of the maternal hypothalamic-pituitary-adrenal axis during pregnancy and lactation. J Neuroendocrinol.

[34] Dickens MJ, Pawluski JL (2018). The HPA axis during the perinatal period: implications for perinatal depression. Endocrinology.

[35] Zorn JV, Schür RR, Boks MP, Kahn RS, Joëls M, Vinkers CH (2017). Cortisol stress reactivity across psychiatric disorders: a systematic review and meta-analysis. Psychoneuroendocrinology.

[36] Kammerer M, Taylor A, Glover V (2006). The HPA axis and perinatal depression: a hypothesis. Arch Womens Ment Health.

[37] O'Connor TG, Tang W, Gilchrist MA, Moynihan JA, Pressman EK, Blackmore ER (2014). Diurnal cortisol patterns and psychiatric symptoms in pregnancy: short-term longitudinal study. Biol Psychol.

[38] Glynn LM, Davis EP, Sandman CA (2013). New insights into the role of perinatal HPA-axis dysregulation in postpartum depression. Neuropeptides.

[39] Glynn LM, Sandman CA (2014). Evaluation of the association between placental corticotrophin-releasing hormone and postpartum depressive symptoms. Psychosom Med.

[40] Pennell KD, Woodin MA, Pennell PB (2015). Quantification of neurosteroids during pregnancy using selective ion monitoring mass spectrometry. Steroids.

[41] Luisi S, Petraglia F, Benedetto C (2000). Serum allopregnanolone levels in pregnant women: changes during pregnancy, at delivery, and in hypertensive patients. J Clin Endocrinol Metab.

[42] Paoletti AM, Romagnino S, Contu R (2006). Observational study on the stability of the psychological status during normal pregnancy and increased blood levels of neuroactive steroids with GABA-A receptor agonist activity. Psychoneuroendocrinology.

[43] Bloch M, Schmidt PJ, Danaceau M, Murphy J, Nieman L, Rubinow DR (2000). Effects of gonadal steroids in women with a history of postpartum depression. Am J Psychiatry.

[44] Osborne LM, Betz JF, Yenokyan G, Standeven LR, Payne JL (2019). The role of allopregnanolone in pregnancy in predicting postpartum anxiety symptoms. Front Psychol.

[45] Stuebe AM, Grewen K, Meltzer-Brody S (2013). Association between maternal mood and oxytocin response to breastfeeding. J Womens Health (Larchmt).

[46] Skrundz M, Bolten M, Nast I, Hellhammer DH, Meinlschmidt G (2011). Plasma oxytocin concentration during pregnancy is associated with development of postpartum depression. Neuropsychopharmacology.

[47] Jobst A, Krause D, Maiwald C (2016). Oxytocin course over pregnancy and postpartum period and the association with postpartum depressive symptoms. Arch Womens Ment Health.

[48] Albacar G, Sans T, Martín-Santos R (2010). Thyroid function 48h after delivery as a marker for subsequent postpartum depression. Psychoneuroendocrinology.

[49] Le Donne M, Settineri S, Benvenga S (2012). Early pospartum alexithymia and risk for depression: relationship with serum thyrotropin, free thyroid hormones and thyroid autoantibodies. Psychoneuroendocrinology.

[50] Lasley SM, Gilbert ME (2011). Developmental thyroid hormone insufficiency reduces expression of brain-derived neurotrophic factor (BDNF) in adults but not in neonates. Neurotoxicol Teratol.

[51] Kuijpens JL, Vader HL, Drexhage HA, Wiersinga WM, van Son MJ, Pop VJ (2001). Thyroid peroxidase antibodies during gestation are a marker for subsequent depression postpartum. Eur J Endocrinol.

[52] Sylvén SM, Elenis E, Michelakos T, Larsson A, Olovsson M, Poromaa IS, Skalkidou A (2013). Thyroid function tests at delivery and risk for postpartum depressive symptoms. Psychoneuroendocrinology.

[53] Li D, Li Y, Chen Y (2019). Neuroprotection of reduced thyroid hormone with increased estrogen and progestogen in postpartum depression. Biosci Rep.

[54] Pedersen C, Leserman J, Garcia N, Stansbury M, Meltzer-Brody S, Johnson J (2016). Late pregnancy thyroid-binding globulin predicts perinatal depression. Psychoneuroendocrinology.

[55] Wesseloo R, Kamperman AM, Bergink V, Pop VJ (2018). Thyroid peroxidase antibodies during early gestation and the subsequent risk of first-onset postpartum depression: a prospective cohort study. J Affect Disord.

[56] Groer MW, Vaughan JH (2013). Positive thyroid peroxidase antibody titer is associated with dysphoric moods during pregnancy and postpartum. J Obstet Gynecol Neonatal Nurs.

[57] Raison CL, Capuron L, Miller AH (2006). Cytokines sing the blues: inflammation and the pathogenesis of depression. Trends Immunol.

[58] Kendall-Tackett K (2007). A new paradigm for depression in new mothers: the central role of inflammation and how breastfeeding and anti-inflammatory treatments protect maternal mental health. Int Breastfeed J.

[59] Corwin EJ, Johnston N, Pugh L (2008). Symptoms of postpartum depression associated with elevated levels of interleukin-1 beta during the first month postpartum. Biol Res Nurs.

[60] Liu H, Zhang Y, Gao Y, Zhang Z (2016). Elevated levels of Hs-CRP and IL-6 after delivery are associated with depression during the 6 months post partum. Psychiatry Res.

[61] Bränn E, Fransson E, White RA (2020). Inflammatory markers in women with postpartum depressive symptoms. J Neurosci Res.

[62] Wójcik J, Dudek D, Grabowska M (2006). Antepartum/postpartum depressive symptoms and serum zinc and magnesium levels. Pharmacol Rep.

[63] Roomruangwong C, Kanchanatawan B, Sirivichayakul S, Mahieu B, Nowak G, Maes M (2017). Lower serum zinc and higher crp strongly predict prenatal depression and physio-somatic symptoms, which all together predict postnatal depressive symptoms. Mol Neurobiol.

[64] Christesen HT, Falkenberg T, Lamont RF, Jørgensen JS (2012). The impact of vitamin D on pregnancy: a systematic review. Acta Obstet Gynecol Scand.

[65] Brandenbarg J, Vrijkotte TG, Goedhart G, van Eijsden M (2012). Maternal early-pregnancy vitamin D status is associated with maternal depressive symptoms in the Amsterdam Born Children and Their Development cohort. Psychosom Med.

[66] Gur EB, Gokduman A, Turan GA (2014). Mid-pregnancy vitamin D levels and postpartum depression. Eur J Obstet Gynecol Reprod Biol.

[67] Robinson M, Whitehouse AJ, Newnham JP (2014). Low maternal serum vitamin D during pregnancy and the risk for postpartum depression symptoms. Arch Womens Ment Health.

[68] Ogiji J, Rich W (2022). An exploratory study of vitamin D levels during pregnancy and its association with postpartum depression. Psy Com.

